# Association of Serum Lipid Level with Meibum Biosynthesis and Meibomian Gland Dysfunction: A Review

**DOI:** 10.3390/jcm11144010

**Published:** 2022-07-11

**Authors:** Young-Sik Yoo, Sun-Kyoung Park, Ho-Sik Hwang, Hyun-Seung Kim, Reiko Arita, Kyung-Sun Na

**Affiliations:** 1 Department of Ophthalmology, College of Medicine, The Catholic University of Korea, Seoul 06591, Korea; theblue07@naver.com (Y.-S.Y.); sunkyoungpark712@gmail.com (S.-K.P.); huanghs@catholic.ac.kr (H.-S.H.); sara514@catholic.ac.kr (H.-S.K.); 2 Itoh Clinic, Saitama 337-0042, Japan; ritoh@za2.so-net.ne.jp; 3 Department of Ophthalmology, Keio University, Tokyo 160-8582, Japan

**Keywords:** serum lipid level, dyslipidemia, hypercholesterolemia, meibum biosynthesis, meibomian gland dysfunction, blepharitis

## Abstract

The primary role of meibomian glands (MGs) is to actively synthesize and secret lipids and proteins spread onto the tear film, and the glandular lipids promote tear stability, prevent evaporation, and reduce friction. Meibomian gland dysfunction (MGD) is the leading cause of dry eye disease and one of the most common ophthalmic problems worldwide. MGs are densely innervated and regulated by hormones and growth factors. However, since the polar and nonpolar lipids are produced through processes in MGs that are not completely understood, a relevant question has been raised: Would the altered systemic lipids metabolism affect the physiology and structure of MGs? This review introduces the recent update regarding the relationships between serum lipid and MGD in clinical and basic research while providing answers to this question. A causal relationship remains to be established; however, serum lipid level or dyslipidemia may be related to MGD directly or indirectly, or both. Further studies are warranted to establish the role of serum lipid level and meibocyte differentiation/maturation and lipid synthesis.

## 1. Introduction

Meibomian glands (MGs) are large sebaceous glands located in the tarsal plates of the eyelids. These are the primary source of the tear film lipid meibum in the tear film, which consists of an aqueous layer and a superficial lipid layer [1]. The tear film lipid layer (TFLL) is ~40 nm thin and preserves a clear and smooth anterior surface, slows tear evaporation, and forms a barrier against microbial agents and organic matter [2,3]. MGs are densely innervated and their modulation of the development, maturation, lipogenesis, and secretion of meibum is orchestrated by hormones and growth factors [1]. Nerve fibers located in the interstitium of MGs and isolated nerve endings surrounding the duct system suggest the influence of the nervous system as well [4]. While blinking, these glands actively synthesize and secrete lipids and proteins onto the lid margin during muscular contractures [5].

**Figure 1:**
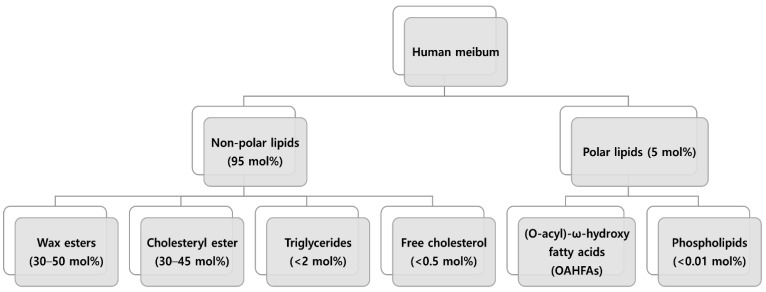
Lipid composition of human meibum.

The secretory acini of the MGs contain meibocytes [1,6]. As these cells mature, they undergo a progressive accumulation of lipids in the cytoplasm, shrinkage, and pyknosis [1]. The disintegration of the cell membrane occurs, and the cell contents form a lipid and protein mixture, meibum, which is excreted into the ducts through holocrine activity [1,6]. The International Dry Eye Workshop II reported the meibum is approximately 95% nonpolar and 5% polar lipid [3]. Nonpolar lipids comprising human meibum consist primarily of 30–50 mol% of wax ester [7,8,9], 35–40 mol% of cholesteryl ester [10,11,12], and less than 2% triglycerides (Figure 1) [9]. Its involvement in lipid biosynthesis or uptake in the MGs remains vague. Theoretically, lipid biosynthesis in MGs can be de novo in acinar cells or through lipid uptake from the bloodstream [1]. Recent studies supporting de novo synthesis reported that synthetic enzymes and hormones regulate the final products [10,11,12]. However, there are few studies regarding the bloodstream uptake of lipid into MGs, which warrants further investigation. This uptake would lead to the alteration of the meibum composition according to variation in serum lipid levels with diet, medication, and systemic condition.

Disorders of meibomian gland function, which are referred to as meibomian gland dysfunction (MGD), are defined as a chronic, diffuse abnormality of the MGs, characterized by terminal duct obstruction and/or qualitative or quantitative changes in the glandular secretion, which may result in the alteration of ocular surface health and homeostasis [13]. A dysfunctional TFLL is believed to be a major underlying reason for the development of dry eye disease, and recent studies have suggested that the specific TFLL lipids—namely, O-acyl-ω-hydroxy fatty acids (OAHFAs) and diesters (DiEs)—may play a role in the pathogenesis [12,14]. MGD has been described for several decades and is now accepted as a discrete disease; however, its etiology as a systemic and ocular disease is yet to be fully investigated. Dyslipidemia is defined as abnormal amounts of lipids and lipoprotein in the blood [15]. Dyslipidemia is characterized by an increased level of total cholesterol (TC), low-density lipoprotein (LDL), and triglyceride (TG), as well as a decreased level of high-density lipoprotein (HDL) in the blood [15]. It is one of the major modifiable risk factors in cardiovascular diseases [16]. Given that lipid biosynthesis occurs in MGs, the question arises whether the systemic lipid metabolism abnormality, dyslipidemia, may be associated with MGD. However, little is understood about the role of systemic serum lipid level or dyslipidemia in meibum production and MG function. This review provides an update on the association between systemic serum lipid and MGD. 

## 2. Sources and Methods of Literature Search

An initial scientific literature search was conducted on 1 October 2021 using the PubMed database. Search filters included a publication date between 1 January 2011 and 30 August 2021.

The following keywords were used in PubMed research as MeSH terms: “meibomian gland dysfunction” and “dyslipidemias”. Since the term “dyslipidemia” covers various lipid profiles, including increased blood levels of cholesterol, triglycerides, or lipoproteins, we used multiple terms during the literature search, such as “elevated cholesterols”, “hypercholesteremia”, “triglyceride”, “high-density lipoproteins”, and “low-density lipoproteins”. In addition, use various versions of the term “meibomian gland dysfunction” to prevent the loss of relevant articles, such as “MG dysfunction”, “MGD patients”, “MGD subtype”, or “Tarsal glands”.

After the initial research, we retrieved 626 articles. We screened the papers sequentially in the following order: titles, abstracts, and full-text. The reference list of each article was also reviewed to identify other relevant papers. Three reviewers (Y.-S.Y., S.-K.P., and K.-S.N.) independently determined the eligibility of each article. The articles satisfying the specified criteria were included. 

We used the Newcastle–Ottawa Scale (NOS), a tool widely used for assessing the risk of bias in nonrandomized observational studies, to assess the quality of included articles. The NOS consists of eight questions categorized into three groups: selection, comparability, and ascertainment of either the exposure or outcome of interest for case–control or cohort studies, respectively. Each study can be awarded a maximum of one star for each numbered item within the selection and exposure/outcome categories. For the comparability category, a maximum of two stars was given. As a result, the highest quality studies can be awarded up to nine stars. For interpretation, three or four stars in the selection category, one or two stars in the comparability domain, and two or three stars in the exposure/outcome category were regarded as “good quality” [17,18]. After performing a NOS-based qualitative assessment, we included articles with a low-risk bias in our manuscript.

**Figure 2:**
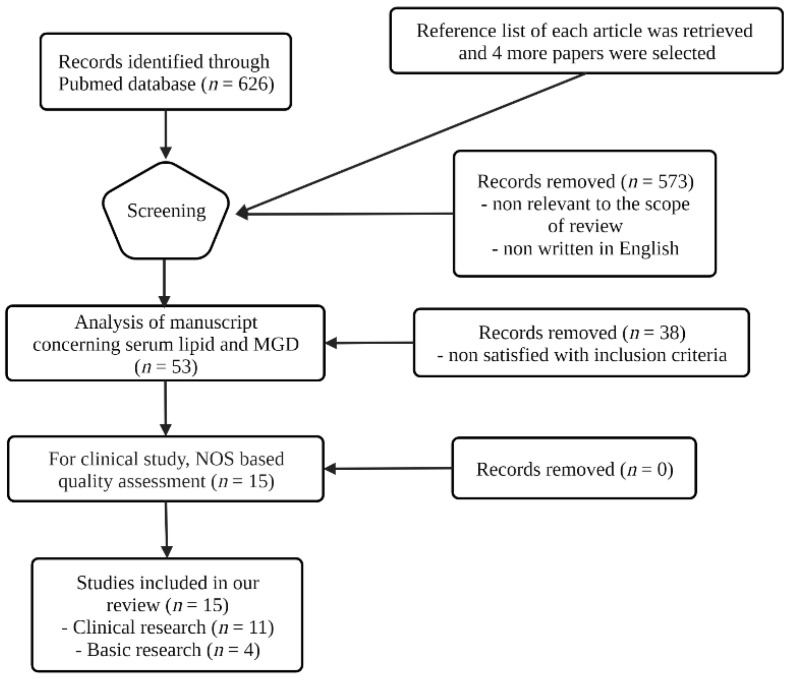
Flow diagram of the literature search and selection for plasma lipid and MGD.

After screening, 573 papers were excluded as they were not relevant to the scope of the review or were not written in English. Based on established inclusion criteria, we excluded 38 papers. Most excluded articles focused on associations between serum lipid and systemic disease other than MGD (cardiovascular disease, diabetes, metabolic syndrome, etc.). After performing a NOS-based quality assessment, we included a final pool of 15 articles on serum lipid levels and MGD. Among the 15 remaining articles, 11 were clinical, and 4 were basic research studies. In addition, we rated each clinical article using the grading systems for assessing the quality of evidence. Citations and full-text papers were exported to Endnote 20 citation manager. The search strategy and results from the listed databases are summarized in a flowchart (Figure 2).

As this review focused on previous clinical studies regarding the association between serum lipid levels and meibomian gland dysfunction (MGD), the NOS tool was not used in the description of previous studies (Section 3.2).

## 3. Research Results from Previous Studies

### 3.1. Clinical Research of Serum Lipid and MGD

Among the clinical research articles that were included in the final selection, nine focused on the association between serum lipid level and MGD, and two focused on the medical treatment of MGD. Among these, seven were case–control studies (Level 3), two were cross-sectional studies (Level 3), one was a prospective cohort study (Level 2), and another one was a randomized controlled trial (Level 1).

Nine of the selected clinical research articles investigated the association between serum lipid levels and MGD [19,20,21,22,23,24,25,26,27]. All nine articles showed evidence of Level 3 as a cross-sectional case–control study. 

Six of the nine articles [20,21,22,23,24,25] compared the mean serum lipid level and the prevalence of patients with dyslipidemia between patients with MGD and controls. Most studies showed a higher prevalence of abnormal serum lipid profiles among patients with MGD than controls; however, there were also controversial results as well.

Compared with the rest of the studies [20,21,22,23,24] with a relatively small number of subjects (58 to 136 subjects), Ha et al. [25] conducted a much larger cohort study in which authors employed age and sex-matched propensity score matching using normal control patients from the National Health and Nutrition Examination Survey (NHANES). 

Except for two recently published articles [24,25], the study by Pinna et al. [20], Braich et al. [21], Chen et al. [22], and Irfan et al. [23] performed multivariate regression analysis to analyze risk factors for MGD, and the results were varied. 

The remaining three articles [19,26,27] investigated the correlation between the severity of MGD and serum lipid level. The prevalence of dyslipidemia tended to increase as the severity of MGD increased. However, all of them did not perform a multivariate regression analysis to analyze risk factors for moderate to severe MGD.

Table 1 shows a summary of the nine above-mentioned clinical research articles concerning the association between serum lipid levels and MGD and compares each article in terms of design, number of participants, date of publication, and results.

We found two clinical research articles that focused on the effect of serum lipid regulating medical treatments on MGD authored in the last 10 years. One was a randomized, double-masked study (Level 1), while the other was a prospective, nonrandomized study (Level 2). Table 2 shows a summary of the two clinical research articles concerning the effect of serum lipid regulating medical treatments on MGD in terms of design, number of par-ticipants, date of publication, and results.

Essential fatty acid supplementation has been shown to significantly improve the serum lipid profile of patients with dyslipidemia [28,29,30,31]. In addition, a significant increase in the saturated fatty acid content of meibum was observed in patients with MGD supplemented with diets rich in omega-3 fatty acids [32]. Moreover, some studies have shown that omega-3 fatty acid supplements improve the clinical symptoms and signs of dry eye or reduce eyelid margin inflammation [33,34,35,36,37]. Eicosapentaenoic acid (EPA), an omega-3 fatty acid, has been shown to induce meibocyte differentiation through PPARγ activation—a process characterized by cell cycle exit, de novo, and transported lipid accumulation in the endoplasmic reticulum, and autophagy [38]. Statins, which are used in the treatment of dyslipidemia, are composed of HMG–CoA reductase inhibitors, which block the biosynthesis of cholesterol. Considering the pharmacologic mechanisms of omega-3 fatty acids and statins, evaluating MGD in patients taking these medications may be useful in investigating the association between MGD and serum lipid alteration [39].

Oleñik et al. [37] studied the effectiveness of omega-3 fatty acids in improving the symptoms and signs of MGD, compared with the placebo group. This study conducted randomized and double-blind trials for a duration of 3 months, and 61 participants with symptomatic MGD were enrolled and randomly assigned to the omega-3 treatment (*n* = 33) and placebo groups (*n* = 31). All participants were required to apply a warm compress for 5 min and scrub their eyes with diluted baby shampoo. The changes in MG expression and lid margin inflammation were assessed at the follow-up visits 1, 2, and 3 months after the initiation of treatment. The mean lid margin inflammation and MG expressibility presented improvement from baseline only in the omega-3 treatment group. 

These results were consistent with the prospective randomized placebo-controlled masked trial study by Macsai [32] in 2008. In this study, omega-3 essential fatty acid supplements were shown to improve clinical symptoms and signs of MGD, as well as the changes in the meibum content. This study suggested two hypotheses that may explain how supplementation with omega-3 essential fatty acids can alleviate MGD. The first hypothesis stated that the metabolism of omega-3 could inhibit the metabolism of omega-6 and, subsequently, fatty acid may decrease the inflammation of the eyelid. The second proposed hypothesis stated that it may change the fatty acid composition, thus leading to changes in lipid properties of meibum.

The prospective and nonrandomized study by Wu et al. [40] investigated the possible association of dyslipidemia and its treatment with MG morphologic changes using meibography. In total, 98 participants were divided into two groups: 85 participants in the statin group and 13 participants in the nonstatin group. The results showed statistically significant increases in the total and upper eyelid meiboscores, lid margin abnormality scores, and deterioration in meibum quality in the statin group during the follow-up visits. Moreover, similar changes in the upper eyelid meiboscores and meibum quality were also observed in the nonstatin group. This study revealed that MG atrophy continued to progress in both groups. As a result of these investigations, the authors suggested that regulating the serum lipid level might be beneficial only during the early stage of MGD without any obstruction. 

In contrast, the Australian Blue Mountains Eye Study III (BMES III) cohort study [41], a large population-based retrospective analysis consisting predominantly of Caucasian Australians aged over 59 years, showed that hypercholesterolemia—as well as serum LDL and HDL levels—had no significant association with DED symptoms, such as dryness, grittiness, itchiness, discomfort, or watering. However, oral statin therapy was associated with an increased risk of one or more moderate to severe symptoms of DED. In this study, unlike other previous studies, the outcome measure was the presence of DED symptoms rather than the clinical diagnosis of DED or MGD. Moreover, since DED symptoms due to MGD, aqueous deficiency, or mixed etiology were not differentiated in the study, it is difficult to conclude that these findings postulate a significant correlation between oral statin therapy and MGD.

### 3.2. Basic Research on Serum Lipids and MGD

To evaluate the pathophysiology of MGD, several methods for collecting and culturing cells have been introduced using MGs from rabbits [42], mice [43,44], and humans [45]. Since harvesting human meibomian gland epithelial cells (HMGECs) were first introduced as a cell type for evaluating MG epithelial cell activity, including lipid profiles, several factors including serum, azithromycin, omega-3, and -6 fatty acids, brimonidine, peroxisome proliferator activator receptor-γ (PPARγ) agonist, and rosiglitazone have been revealed as molecules that promoted lipid production and differentiation from HMGECs [44,46,47,48,49,50,51,52,53]. Immortalized HMGECs are a valuable resource for certain types of basic regulatory research; however, they are not suited to system-Level 1nvestigations, such as meibum composition, which requires input from multiple stages of MG development, ductal enzymes, and other factors unavailable in vitro [54,55].

Various animal models for MGD have also been proposed by researchers. Key genes for lipid biosynthesis have been demonstrated to make changes in the meibum lipid profiles and cause pathologic abnormality in the ocular surface and MG physiology and morphology [39]. *Elovl1* [56] and *Elovl3* [57] genes, or inhibition of FA ω-oxidation by inactivation of *Cyp4F39* [58], or inactivation of *Awat2* [59,60], led to equally massive changes in meibum lipid profiles and MG and ocular surface physiology and morphology in mutant mice. These gene mutations described the relation between FA elongation, oxidation, and esterification into WE, MG lipid homeostasis, and various ocular pathologies. Recently, Soat1/SOAT1 [11] was reported as the gene causing the elimination of CE and Chl-OAHFA derived from MGs.

The association of serum lipid changes, daily diet affecting serum lipid composition, and MGD have been evaluated through in vivo animal studies [61]. Apolipoprotein E knockout (ApoE^−/^^−^) mice [62] with high increased total serum cholesterol levels demonstrated that obstructive MGD and hyperlipidemia were closely related by confirming MG dropout and disordered acini and ducts in the upper and lower eyelids [63]. HR-1 hairless mice fed a limited lipid diet (HR-AD, a special diet with limited lipid content) were effective in inducing posterior blepharitis around the eyelid margin, plugging orifices, and toothpaste-like meibum [64]. In addition, the C57BL/6 mouse model fed a high-fat diet showed ocular surface damages that can be caused by MGD, such as decreased tear production, notable Oregon green dextran staining, distinct conjunctival goblet cell loss, and squamous metaplasia [65].

Over the years, Burns et al. investigated the relation between dyslipidemia and MGD with a diet-induced obesity mouse model [66]. They obtained obese mice with a high-fat diet and found corneal dysfunction such as both an increase in corneal inflammatory mediators and a decrease in corneal nerve density before the development of sustained hyperglycemia [67]. Additionally, dyslipidemia in their diet-induced obesity mouse model was found to be accompanied by the alteration in both meibum composition and MG structure [68]. The level of the lipid species with saturated fatty acids was increased in meibum, and MG hypertrophy was confirmed with meibography in their animal study [68]. Table 3 shows a summary of basic research articles concerning the pathophysiology of MGD in relation to dyslipidemia using an animal model.

## 4. Discussion

The association between serum lipids and MGD can be analyzed in direct and indirect ways. Altering the lipid composition in the blood Level 1n dyslipidemia and MGD has shown conflicting results that cholesterol and LDL, and HDL may or may not be related to MGD [19,20,21,22,23,24,25,26,27]. There is no Level 1 evidence of the association between MGD and serum lipid levels. Some previous case–control studies reported that elevated TC, TG, or LDL was associated with MGD [20,21,22,23], whereas some others reported no significant association [19,24,25,26,27]. HDL is known as a favorable lipoprotein in cardiovascular function; however, some studies have shown that high HDL may be associated with MGD [20,21,25]. In addition, medication or dietary supplements that alter the lipid composition may act as a useful clue for the association between serum lipid and MGD. Such an uptake would lead to changes in the meibum composition according to diet status; medication use, such as statin or omega-3, 6 fatty acid; or lipid profile in serum. One Level 1 study showed that dietary supplementation with omega-3 fatty acids resulted in a decrease in the serum ratios of omega-6 to omega-3 and showed improvements in overall Ocular Surface Disease Index score, tear breakup time, and meibum score [32]. Moreover, the study demonstrated an induced change in the fatty acid saturation content in meibum as a result of dietary supplementation with omega-3 fatty acids. This study suggested that altered serum lipid levels would affect the lipid biosynthesis in acinar cells and the final product either directly or indirectly. In contrast, the results of a prospective nonrandomized study to evaluate the possible association between treating dyslipidemia with statins and MGs morphologic changes revealed that MGD progression occurred despite lipid control [40]. Most studies on MGD and dyslipidemia excluded subjects taking lipid-lowering medication, and therefore, the association between statins and MGD has not been shown, although they were eliminated as a potential confounder. Well-designed randomized controlled studies with longer follow-ups and potentially providing Level 1 evidence are recommended to confirm the association between dyslipidemia, statin use, and MGD.

There are several clinical studies evaluating the relationship between serum lipid levels and MGD, but few classify as Level 1 studies; most reports available for this review included retrospective cohort studies with limited numbers and inclusion criteria. Some studies have been conducted on animal models and cultured meibocytes [45,46,47,48,49,50,51,52,53,56,57,59,60,63,64,65,66,68], but their findings were not conclusive. Lipidomic studies may aid in identifying a link between MGD and serum/plasma lipid species, since prostaglandins and leukotrienes are eicosanoids derived from arachidonic acid and related polyunsaturated fatty acids, participating in both normal homeostasis and inflammatory conditions [69]. However, to our knowledge, no relevant studies have reported specific lipid profiles for free FA, glycerophospholipids, sphingolipids, or glycerolipids in the human serum or plasma. Genetic manipulation animal models, such as ApoE^−/−^ mice, show a marked increase in TC in serum mimic the obstructive MGD, including MGs orifice plugging duct dilatation and acinar deformation [63]. Diet-induced obesity models where mice develop dyslipidemia were expected to be useful tools for investigating the effects of dyslipidemia and MGD in future studies [64,66,68].

There were several hypotheses on why serum lipid levels may alleviate or aggravate MG function and result in DED symptoms. The first hypothesis was based on systemic inflammation leading to changes in lipid metabolism aimed at decreasing the toxicity of a variety of harmful agents and tissue repair by redistributing nutrients to cells [70]. As MGD and DED are believed to be closely related to inflammation, alleviation or aggravation of systemic inflammatory status may affect both lipid metabolism systemically, in addition to the eyelids and MGs [71,72,73]. Thus, dyslipidemia and MGD may share common pathogenesis regarding inflammation. In this context, we can assume that DED may be a result of both dyslipidemia and MGD. There are studies supporting that dyslipidemia and MGD are closely linked, although the causal relationship is yet to be investigated. Previously, in an animal study, desiccating stress was shown to affect the meibum maturation process and thus alter the lipid/protein composition of meibum [74]. Changes in meibum quality may lead to MGD by further affecting tear instability and ocular surface inflammation. In addition, PPARs may be a connecting link between dyslipidemia and MGD. PPARs are involved in the metabolic regulation of lipid and lipoprotein levels, such as triglycerides, blood glucose, and abdominal obesity, and are now widely accepted as a valuable therapeutic target in the regulation of metabolic homeostasis [75]. Among the subtypes of PPARs, PPARγ has been suggested as a master regulator of meibocyte differentiation and function [75]. Jester et al. reported that aging MGs showed altered PPARγ expression and decreased meibocyte differentiation and lipid biosynthesis in human tissue samples and mouse models [76]. Systemically, PPARγ is mainly expressed in adipocytes and plays a major role in cell differentiation and energy metabolism [75]. Pioglitazone, which is an agonist of PPARγ, has demonstrated beneficial effects in reducing TG and increasing HDL levels [77]. These findings support the premise that altering PPARγ expression may result in changes in lipid composition in serum levels and also MG differentiation and meibum synthesis.

The influence of the systemic lipid level on the meibum synthesis in the MGs is still underinvestigated. Epidemiological studies revealed that there are racial differences in the prevalence of MGD [78]. This implies that dietary habits, energy supply, food complexity, and genetic vulnerability play a role in the prevalence of MGD. These complex and co-causative factors of the MGD pathogenesis would affect lipid uptake systemically, as well as the biosynthesis of meibum in MGs. Additional information should be considered regarding the association between serum lipid and MGD. Generally, MGD is classified into three forms: hypersecretory, hyposecretory, and obstructive, with the latter form being considered the most common [2,13]. Each subtype may or may not have similar pathogenesis, which might be linked to serum lipid composition. In MGD, the hyperkeratinization of ductal epithelium, the increased viscosity of meibum, and terminal duct obstruction may affect each other or occur separately. The obstruction is affected by multiple causes including age, sex, and hormones. Based on the association between dyslipidemia and DED suggested by previous studies, the underlying DED may result in ocular surface inflammation and hyperkeratinization. In contrast, there is also evidence that the glands may undergo acinar atrophy with aging, inflammation, and infection [1]. Dyslipidemia may regulate the differentiation and renewal of meibocytes that directly impact meibum composition, possibly by altering PPARγ expression and localization, as suggested by Jester et al. [44,46,79]. A direct comparison between the clinical studies included in this review is difficult because of the different subjects enrolled. Therefore, a more detailed classification of MGD patients is needed in further studies.

## 5. Conclusions

As shown in this review, serum lipid level or dyslipidemia may be related to MGD directly or indirectly, or both. Dyslipidemia, inflammation, co-existing or underlying DED, decreased proliferative properties of meibocyte and acinar atrophy, and alteration of biosynthesis and the changing composition of meibum are interrelated in the pathogenesis of MGD. Notably, there are some studies that show that high HDL, although beneficial for atherosclerosis and metabolic syndrome, may affect MG function and DED negatively, which warrants further research. Continued efforts to conduct research studies, particularly randomized controlled trials, are necessary to establish the role of serum lipid level and meibocyte differentiation/maturation, and lipid synthesis.

## Figures and Tables

**Table 1:** Summary of clinical research articles concerning association between plasma lipid levels and MGD.

	Pinna et al. [20]	Braich et al. [21]	Chen et al. [22]	Irfan et al. [23]				Mussi et al. [24]		Ha et al. [25]			Guliani et al. [19]	Bukhari et al. [26]	Tulsyan et al. [27]
**Level of evidence**	Level 3	Level 3	Level 3	Level 3				Level 3		Level 3			Level 3	Level 3	Level 3
**Design**	Case-control study	Case-control study	Case-control study	Case-control study				Case-control study		Case-control study			Case-control study	Case-control study	Case-control study
**Number of participants (case/control)**	60/63	109/115	199/89	58/58				163/136		95/475			90/90	132/104	237/163
**Year**	2013	2016	2017	2020				2021		2021			2018	2013	2021
**Results**															
**Mean serum lipid Level 1n patients with MGD (compared to those without MGD)**													**Mean serum lipid Level 1n patients with moderate to severe MGD (compared to mild MGD)**		
**Total cholesterol**	Higher ^†^	Higher	Higher	N/A				Lower (NS)		Lower (NS)			N/A	N/A	N/A
**LDL**	Higher	Higher ^†^	Higher	N/A				Higher (NS)		Lower			N/A	N/A	N/A
**TG**	Higher	Higher	Higher	N/A				Higher (NS)		Lower (NS)			N/A	N/A	N/A
**HDL**	Higher	Higher	Lower	N/A				Lower (NS)		Higher			N/A	N/A	N/A
Prevalence of dyslipidemia * among patients with MGD (compared to those without MGD)													Prevalence of dyslipidemia * in patients with moderate to severe MGD (compared to mild MGD)		
**Gender**								**Female**	**Male**						
**Age**										**<45**	**45–64**	**≥65**			
**Total cholesterol**	N/A	Higher	N/A	Higher				Lower (NS)	Higher (NS)	-	-	-	Higher	N/A	Higher (NS)
**LDL**	N/A	Higher	N/A	Higher				Lower (NS)	Higher (NS)	-	Higher	Higher	Higher	Higher (NS)	Higher
**TG**	N/A	Higher	N/A	Higher				N/A	N/A	-	-	-	Higher	Higher (NS)	Higher (NS)
**HDL**	N/A	Lower (NS)	N/A	Higher (NS)				Lower (NS)	Higher (NS)	-	Higher	-	Higher	N/A	Higher (NS)
**Factors impacting development of MGD**															
**MGD grade**				**1**	**2**	**3**	**4**								
**Total cholesterol**	V	V	-	-	V	V	-	N/A		N/A			N/A	N/A	N/A
**LDL**	V	V	V	-	-	-	-	N/A		N/A			N/A	N/A	N/A
**TG**	-	V	V	-	-	V,	V	N/A		N/A			N/A	N/A	N/A
**HDL**	V	-	-	-	-	-	-	N/A		N/A			N/A	N/A	N/A

Abbreviations: LDL, low-density lipoproteins; TG, triglyceride; HDL, high-density lipoproteins; MGD, meibomian gland dysfunction; NS, not significant; N/A, not available. * Dyslipidemia was defined as a fasting total cholesterol level of ≥200 mg/dL, LDL ≥ 130 mg/dL, triglycerides ≥ 150 mg/dL, and HDL ≤ 40 mg/dL in Guliani et al., Dao et al., Pinna et al., Braich et al., and the studies by Irfan and Mussi. In the study by Bukhari et al., dyslipidemia was defined as a fasting total cholesterol level of >200 mg/dL, triglyceride > 200 mg/dL, LDL > 130 mg/dL, and HDL < 40 mg/dL. In the study by Chen et al., dyslipidemia was defined as fasting total cholesterol level of ≥240 mg/dL, LDL ≧ 160 mg/dL, and HDL < 40 mg/dL. In the study by Ha et al., dyslipidemia was defined as fasting total cholesterol level of ≥200 mg/dL, triglyceride > 143 mg/dL, LDL ≧ 140 mg/dL, and HDL < 40 mg/dL. In the study by Tulsyan, the criteria for dyslipidemia were not mentioned. ^†^ Abnormal serum lipid level.

**Table 2:** Summary of clinical research articles concerning the effect of serum lipid regulating medical treatments on MGD.

Author	Year	Level of Evidence	Study Design	Number of Patients		Results			
				Case	Control	Lid Margin Abnormality	MG Expressibility	MG Quality	MG Morphology
Oleñik et al. [37]	2013	Level 1	Randomized, double-masked study	Omega-3 treatment group (*n* = 33)	Placebo group (*n* = 31)	Improved	Improved		
Wu et al. [39]	2020	Level 2	Prospective, nonrandomized study	Statin treatment group (*n* = 85)	Life style intervention group (*n* = 13)	Deteriorated		Deteriorated	Deteriorated

Abbreviations: MG, meibomian gland.

**Table 3:** Summary of basic research articles concerning pathophysiology of MGD in relation to dyslipidemia using animal models.

Author	Year	Animal Model	MG Features	Other Defects
Bu et al. [63]	2019	Apolipoprotein E knockout mice (ApoE(^−/−^))	Plugging of the meibomian gland orifice, duct dilation, and heteromorphic acinar morphology.	Present punctate corneal staining and signs of corneal damage
Miyake et al. [64]	2016	HR-1 hairless mice fed an HR-AD diet (a special diet with limited lipid content)	Plugging of the meibomian gland orifice and toothpaste-like meibum.	-
Osae et al. [68]	2020	C57BL/6 mice fed a high-fat diet	Hypertrophic change in meibomian gland and increased lipid saturation in meibum.	-

## Data Availability

Not applicable.

## References

[1] Knop E., Knop N., Millar T., Obata H., Sullivan D.A. (2011). The international workshop on meibomian gland dysfunction: Report of the subcommittee on anatomy, physiology, and pathophysiology of the meibomian gland. Investig. Ophthalmol. Vis. Sci..

[2] Nichols K.K., Foulks G.N., Bron A.J., Glasgow B.J., Dogru M., Tsubota K., Lemp M.A., Sullivan D.A. (2011). The international workshop on meibomian gland dysfunction: Executive summary. Investig. Ophthalmol. Vis. Sci..

[3] Willcox M.D.P., Argüeso P., Georgiev G.A., Holopainen J.M., Laurie G.W., Millar T.J., Papas E.B., Rolland J.P., Schmidt T.A., Stahl U. (2017). TFOS DEWS II Tear Film Report. Ocul. Surf..

[4] Bründl M., Garreis F., Schicht M., Dietrich J., Paulsen F. (2021). Characterization of the innervation of the meibomian glands in humans, rats and mice. Ann. Anat..

[5] Linton R.G., Curnow D.H., Riley W.J. (1961). The meibomian glands: An investigation into the secretion and some aspects of the physiology. Br. J. Ophthalmol..

[6] Nicolaides N., Kaitaranta J.K., Rawdah T.N., Macy J.I., Boswell F.M., Smith R.E. (1981). Meibomian gland studies: Comparison of steer and human lipids. Investig. Ophthalmol. Vis. Sci..

[7] Butovich I.A. (2009). The Meibomian puzzle: Combining pieces together. Prog. Retin. Eye Res..

[8] Pucker A.D., Nichols J.J. (2012). Analysis of meibum and tear lipids. Ocul. Surf..

[9] Butovich I.A. (2013). Tear film lipids. Exp. Eye Res..

[10] Brown S.H., Kunnen C.M., Duchoslav E., Dolla N.K., Kelso M.J., Papas E.B., Lazon de la Jara P., Willcox M.D., Blanksby S.J., Mitchell T.W. (2013). A comparison of patient matched meibum and tear lipidomes. Investig. Ophthalmol. Vis. Sci..

[11] Butovich I.A., Wilkerson A., Yuksel S. (2021). Depletion of Cholesteryl Esters Causes Meibomian Gland Dysfunction-Like Symptoms in a Soat1-Null Mouse Model. Int. J. Mol. Sci..

[12] Lam S.M., Tong L., Yong S.S., Li B., Chaurasia S.S., Shui G., Wenk M.R. (2011). Meibum lipid composition in Asians with dry eye disease. PLoS ONE.

[13] Nelson J.D., Shimazaki J., Benitez-del-Castillo J.M., Craig J.P., McCulley J.P., Den S., Foulks G.N. (2011). The international workshop on meibomian gland dysfunction: Report of the definition and classification subcommittee. Investig. Ophthalmol. Vis. Sci..

[14] Bland H.C., Moilanen J.A., Ekholm F.S., Paananen R.O. (2019). Investigating the Role of Specific Tear Film Lipids Connected to Dry Eye Syndrome: A Study on O-Acyl-ω-hydroxy Fatty Acids and Diesters. Langmuir.

[15] Klop B., Elte J.W., Cabezas M.C. (2013). Dyslipidemia in obesity: Mechanisms and potential targets. Nutrients.

[16] Nikolic D., Castellino G., Banach M., Toth P.P., Ivanova E., Orekhov A.N., Montalto G., Rizzo M. (2017). PPAR Agonists, Atherogenic Dyslipidemia and Cardiovascular Risk. Curr. Pharm. Des..

[17] Stang A. (2010). Critical evaluation of the Newcastle-Ottawa scale for the assessment of the quality of nonrandomized studies in meta-analyses. Eur. J. Epidemiol..

[18] Peterson J., Welch V., Losos M., Tugwell P. (2011). The Newcastle-Ottawa scale (NOS) for assessing the quality of nonrandomised studies in meta-analyses. Ott. Hosp. Res. Inst..

[19] Guliani B.P., Bhalla A., Naik M.P. (2018). Association of the severity of meibomian gland dysfunction with dyslipidemia in Indian population. Indian J. Ophthalmol..

[20] Pinna A., Blasetti F., Zinellu A., Carru C., Solinas G. (2013). Meibomian gland dysfunction and hypercholesterolemia. Ophthalmology.

[21] Braich P.S., Howard M.K., Singh J.S. (2016). Dyslipidemia and its association with meibomian gland dysfunction. Int. Ophthalmol..

[22] Chen A., Chen H.T., Chen H.C., Chen Y.T., Hwang Y.H., Sun C.C., Hsiao C.H., Ma D.H., Wu W.C., Lai C.C. (2017). Asymptomatic Meibomian Gland Dysfunction and Cardiovascular Disease Risk Factors in a Middle-Aged Population in Taiwan—A Cross-sectional Analysis. Sci. Rep..

[23] Irfan K.S.A., Agrawal A., Singh A., Mittal S.K., Samanta R. (2020). Association of Lipid Profile with Severity of Meibomian Gland Dysfunction. Nepal J. Ophthalmol..

[24] Mussi N., Haque W., Robertson D.M. (2021). The Association Between Risk Factors for Metabolic Syndrome and Meibomian Gland Disease in a Dry Eye Cohort. Clin. Ophthalmol..

[25] Ha M., Song J., Park S., Han K., Hwang H.S., Kim H.S., Arita R., Na K.S. (2021). Relationship between serum lipid level and meibomian gland dysfunction subtype in Korea using propensity score matching. Sci. Rep..

[26] Bukhari A.A. (2013). Associations between the grade of meibomian gland dysfunction and dyslipidemia. Ophthalmic Plast. Reconstr. Surg..

[27] Tulsyan N., Gupta N., Agrawal N. (2021). Risk Factors Associated with Meibomian Gland Dysfunction: A Hospital Based Study. Nepal J. Ophthalmol..

[28] Rajaram S., Haddad E.H., Mejia A., Sabaté J. (2009). Walnuts and fatty fish influence different serum lipid fractions in normal to mildly hyperlipidemic individuals: A randomized controlled study. Am. J. Clin. Nutr..

[29] Carrero J.J., Fonollá J., Marti J.L., Jiménez J., Boza J.J., López-Huertas E. (2007). Intake of fish oil, oleic acid, folic acid, and vitamins B-6 and E for 1 year decreases plasma C-reactive protein and reduces coronary heart disease risk factors in male patients in a cardiac rehabilitation program. J. Nutr..

[30] Demonty I., Chan Y.M., Pelled D., Jones P.J. (2006). Fish-oil esters of plant sterols improve the lipid profile of dyslipidemic subjects more than do fish-oil or sunflower oil esters of plant sterols. Am. J. Clin. Nutr..

[31] Kelley D.S., Siegel D., Vemuri M., Chung G.H., Mackey B.E. (2008). Docosahexaenoic acid supplementation decreases remnant-like particle-cholesterol and increases the (n-3) index in hypertriglyceridemic men. J. Nutr..

[32] Macsai M.S. (2008). The role of omega-3 dietary supplementation in blepharitis and meibomian gland dysfunction (an AOS thesis). Trans. Am. Ophthalmol. Soc..

[33] Sheppard J.D., Singh R., McClellan A.J., Weikert M.P., Scoper S.V., Joly T.J., Whitley W.O., Kakkar E., Pflugfelder S.C. (2013). Long-term Supplementation With n-6 and n-3 PUFAs Improves Moderate-to-Severe Keratoconjunctivitis Sicca: A Randomized Double-Blind Clinical Trial. Cornea.

[34] Korb D.R., Blackie C.A., Finnemore V.M., Douglass T. (2015). Effect of using a combination of lid wipes, eye drops, and omega-3 supplements on meibomian gland functionality in patients with lipid deficient/evaporative dry eye. Cornea.

[35] Malhotra C., Singh S., Chakma P., Jain A.K. (2015). Effect of oral omega-3 Fatty Acid supplementation on contrast sensitivity in patients with moderate meibomian gland dysfunction: A prospective placebo-controlled study. Cornea.

[36] Pinna A., Piccinini P., Carta F. (2007). Effect of oral linoleic and gamma-linolenic acid on meibomian gland dysfunction. Cornea.

[37] Oleñik A., Jiménez-Alfaro I., Alejandre-Alba N., Mahillo-Fernández I. (2013). A randomized, double-masked study to evaluate the effect of omega-3 fatty acids supplementation in meibomian gland dysfunction. Clin. Interv. Aging.

[38] Kim S.W., Rho C.R., Kim J., Xie Y., Prince R.C., Mustafa K., Potma E.O., Brown D.J., Jester J.V. (2020). Eicosapentaenoic acid (EPA) activates PPARγ signaling leading to cell cycle exit, lipid accumulation, and autophagy in human meibomian gland epithelial cells (hMGEC). Ocul. Surf..

[39] Butovich I.A., Bhat N., Wojtowicz J.C. (2019). Comparative Transcriptomic and Lipidomic Analyses of Human Male and Female Meibomian Glands Reveal Common Signature Genes of Meibogenesis. Int. J. Mol. Sci..

[40] Wu K.I., Chen C.Y., Jou T.S., Jimmy Juang J.M., Lu J.Y., Wang I.J. (2020). Effect of 3-Hydroxy-3-Methyl-Glutaryl-Coenzyme A Reductase Inhibitors on the Meibomian Gland Morphology in Patients with Dyslipidemia. Am. J. Ophthalmol..

[41] Ooi K.G., Lee M.H., Burlutsky G., Gopinath B., Mitchell P., Watson S. (2019). Association of dyslipidaemia and oral statin use, and dry eye disease symptoms in the Blue Mountains Eye Study. Clin. Exp. Ophthalmol..

[42] Maskin S.L., Tseng S.C. (1991). Culture of rabbit meibomian gland using collagen gel. Investig. Ophthalmol. Vis. Sci..

[43] Richards S., Schirra F., Sullivan D. (2002). Development of a defined, serum-free culture system for the maintenance of epithelial cells from the mouse meibomian gland. Investig. Ophthalmol. Vis. Sci..

[44] Jester J.V., Potma E., Brown D.J. (2016). PPARγ Regulates Mouse Meibocyte Differentiation and Lipid Synthesis. Ocul. Surf..

[45] Liu S., Hatton M.P., Khandelwal P., Sullivan D.A. (2010). Culture, immortalization, and characterization of human meibomian gland epithelial cells. Investig. Ophthalmol. Vis. Sci..

[46] Kim S.W., Xie Y., Nguyen P.Q., Bui V.T., Huynh K., Kang J.S., Brown D.J., Jester J.V. (2018). PPARγ regulates meibocyte differentiation and lipid synthesis of cultured human meibomian gland epithelial cells (hMGEC). Ocul. Surf..

[47] Liu Y., Kam W.R., Ding J., Sullivan D.A. (2014). Effect of azithromycin on lipid accumulation in immortalized human meibomian gland epithelial cells. JAMA Ophthalmol..

[48] Han X., Liu Y., Kam W.R., Sullivan D.A. (2018). Effect of brimonidine, an α2 adrenergic agonist, on human meibomian gland epithelial cells. Exp. Eye Res..

[49] Hampel U., Krüger M., Kunnen C., Garreis F., Willcox M., Paulsen F. (2015). In vitro effects of docosahexaenoic and eicosapentaenoic acid on human meibomian gland epithelial cells. Exp. Eye Res..

[50] Liu Y., Kam W.R., Ding J., Sullivan D.A. (2014). One man’s poison is another man’s meat: Using azithromycin-induced phospholipidosis to promote ocular surface health. Toxicology.

[51] Ziemanski J.F., Wilson L., Barnes S., Nichols K.K. (2020). Saturation of cholesteryl esters produced by human meibomian gland epithelial cells after treatment with rosiglitazone. Ocul. Surf..

[52] Sullivan D.A., Liu Y., Kam W.R., Ding J., Green K.M., Shaffer S.A., Hatton M.P., Liu S. (2014). Serum-induced differentiation of human meibomian gland epithelial cells. Investig. Ophthalmol. Vis. Sci..

[53] Liu Y., Kam W.R., Sullivan D.A. (2016). Influence of Omega 3 and 6 Fatty Acids on Human Meibomian Gland Epithelial Cells. Cornea.

[54] Asano N., Hampel U., Garreis F., Schröder A., Schicht M., Hammer C.M., Paulsen F. (2018). Differentiation Patterns of Immortalized Human Meibomian Gland Epithelial Cells in Three-Dimensional Culture. Investig. Ophthalmol. Vis. Sci..

[55] Butovich I.A. (2017). Meibomian glands, meibum, and meibogenesis. Exp. Eye Res..

[56] Sassa T., Tadaki M., Kiyonari H., Kihara A. (2018). Very long-chain tear film lipids produced by fatty acid elongase ELOVL1 prevent dry eye disease in mice. FASEB J..

[57] Butovich I.A., Wilkerson A., Bhat N., McMahon A., Yuksel S. (2019). On the pivotal role of Elovl3/ELOVL3 in meibogenesis and ocular physiology of mice. FASEB J..

[58] Miyamoto M., Sassa T., Sawai M., Kihara A. (2020). Lipid polarity gradient formed by ω-hydroxy lipids in tear film prevents dry eye disease. eLife.

[59] Widjaja-Adhi M.A.K., Silvaroli J.A., Chelstowska S., Trischman T., Bederman I., Sayegh R., Golczak M. (2020). Deficiency in Acyl-CoA:Wax Alcohol Acyltransferase 2 causes evaporative dry eye disease by abolishing biosynthesis of wax esters. FASEB J..

[60] McMahon A., Yuksel S., Bhat N., Pham H., Wilkerson A., Butovich I.A. (2020). Inactivation of Awat2 in mice causes loss of wax ester lipids from meibum. Investig. Ophthalmol. Vis. Sci..

[61] Sun M., Moreno I.Y., Dang M., Coulson-Thomas V.J. (2020). Meibomian Gland Dysfunction: What Have Animal Models Taught Us?. Int. J. Mol. Sci..

[62] Osada J., Joven J., Maeda N. (2000). The value of apolipoprotein E knockout mice for studying the effects of dietary fat and cholesterol on atherogenesis. Curr. Opin. Lipidol..

[63] Bu J., Wu Y., Cai X., Jiang N., Jeyalatha M.V., Yu J., He X., He H., Guo Y., Zhang M. (2019). Hyperlipidemia induces meibomian gland dysfunction. Ocul. Surf..

[64] Miyake H., Oda T., Katsuta O., Seno M., Nakamura M. (2016). Meibomian Gland Dysfunction Model in Hairless Mice Fed a Special Diet with Limited Lipid Content. Investig. Ophthalmol. Vis. Sci..

[65] Wu Y., Wu J., Bu J., Tang L., Yang Y., Ouyang W., Lin X., Liu Z., Huang C., Quantock A.J. (2020). High-fat diet induces dry eye-like ocular surface damages in murine. Ocul. Surf..

[66] Osae E.A., Steven P., Redfern R., Hanlon S., Smith C.W., Rumbaut R.E., Burns A.R. (2019). Dyslipidemia and Meibomian Gland Dysfunction: Utility of Lipidomics and Experimental Prospects with a Diet-Induced Obesity Mouse Model. Int. J. Mol. Sci..

[67] Hargrave A., Courson J.A., Pham V., Landry P., Magadi S., Shankar P., Hanlon S., Das A., Rumbaut R.E., Smith C.W. (2020). Corneal dysfunction precedes the onset of hyperglycemia in a mouse model of diet-induced obesity. PLoS ONE.

[68] Osae E.A., Bullock T., Chintapalati M., Brodesser S., Hanlon S., Redfern R., Steven P., Smith C.W., Rumbaut R.E., Burns A.R. (2020). Obese Mice with Dyslipidemia Exhibit Meibomian Gland Hypertrophy and Alterations in Meibum Composition and Aqueous Tear Production. Int. J. Mol. Sci..

[69] Bagga D., Wang L., Farias-Eisner R., Glaspy J.A., Reddy S.T. (2003). Differential effects of prostaglandin derived from omega-6 and omega-3 polyunsaturated fatty acids on COX-2 expression and IL-6 secretion. Proc. Natl. Acad. Sci. USA.

[70] Esteve E., Ricart W., Fernández-Real J.M. (2005). Dyslipidemia and inflammation: An evolutionary conserved mechanism. Clin. Nutr..

[71] Henrich C.F., Ramulu P.Y., Akpek E.K. (2014). Association of dry eye and inflammatory systemic diseases in a tertiary care-based sample. Cornea.

[72] Liew M.S., Zhang M., Kim E., Akpek E.K. (2012). Prevalence and predictors of Sjogren’s syndrome in a prospective cohort of patients with aqueous-deficient dry eye. Br. J. Ophthalmol..

[73] Sullivan D.A., Dana R., Sullivan R.M., Krenzer K.L., Sahin A., Arica B., Liu Y., Kam W.R., Papas A.S., Cermak J.M. (2018). Meibomian Gland Dysfunction in Primary and Secondary Sjögren Syndrome. Ophthalmic Res..

[74] Suhalim J.L., Parfitt G.J., Xie Y., De Paiva C.S., Pflugfelder S.C., Shah T.N., Potma E.O., Brown D.J., Jester J.V. (2014). Effect of desiccating stress on mouse meibomian gland function. Ocul. Surf..

[75] Botta M., Audano M., Sahebkar A., Sirtori C.R., Mitro N., Ruscica M. (2018). PPAR Agonists and Metabolic Syndrome: An Established Role?. Int. J. Mol. Sci..

[76] Nien C.J., Massei S., Lin G., Nabavi C., Tao J., Brown D.J., Paugh J.R., Jester J.V. (2011). Effects of age and dysfunction on human meibomian glands. Arch. Ophthalmol..

[77] Betteridge D.J. (2009). Chicago, periscope and PROactive: CV risk modification in diabetes with pioglitazone. Fundam. Clin. Pharmacol..

[78] Alghamdi Y.A., Mercado C., McClellan A.L., Batawi H., Karp C.L., Galor A. (2016). Epidemiology of Meibomian Gland Dysfunction in an Elderly Population. Cornea.

[79] Jester J.V., Brown D.J. (2012). Wakayama Symposium: Peroxisome proliferator-activated receptor-gamma (PPARγ) and meibomian gland dysfunction. Ocul. Surf..

